# TREM2 Overexpression Attenuates Cognitive Deficits in Experimental Models of Vascular Dementia

**DOI:** 10.1155/2020/8834275

**Published:** 2020-06-12

**Authors:** Qian Wang, Weixia Yang, Jingmei Zhang, Yueran Zhao, Yuzhen Xu

**Affiliations:** ^1^Department of Central Laboratory, Shandong Provincial Hospital Affiliated to Shandong University, Jinan, Shandong Province 250021, China; ^2^Department of Central Laboratory, Taian City Central Hospital, Shandong First Medical University & Shandong Academy of Medical Sciences, Taian, Shandong Province 271000, China; ^3^Department of Neurology, Qingpu Branch of Zhongshan Hospital, Fudan University, Shanghai 201700, China; ^4^Institute of Behavioral Medicine Education, Jining Medical University, Jining, Shandong Province 272067, China; ^5^Department of Neurology, Taian City Central Hospital, Shandong First Medical University & Shandong Academy of Medical Sciences, Taian, Shandong Province 271000, China

## Abstract

Neuroinflammation plays a prominent role in the pathogenesis of vascular dementia (VD). Triggering receptor expressed on myeloid cells 2 (TREM2) is a transmembrane receptor mainly expressed on microglia and has been known for its anti-inflammatory properties during immune response. However, data evaluating the effects of TREM2 in VD are lacking. Therefore, the present study is aimed at investigating the role of TREM2 in VD. In this study, the mouse model of VD was induced by transient bilateral common carotid artery occlusion (BCCAO). We compared the hippocampal gene and protein expressions of TREM2 between the VD mice and sham-operated mice at different time points. The TREM2 mRNA and protein expression levels in the VD mice were higher than those in the sham-operated mice. The cognitive deficits of VD mice were observed in the Morris water maze test. Interestingly, overexpression of TREM2 by intracerebroventricular injection of a lentiviral vector that encoded TREM2 (LV-TREM2) significantly improved the spatial learning and memory and attenuated the hippocampal neural loss in VD mice. Further mechanistic study revealed that overexpression of TREM2 significantly inhibited microglia M1 polarization by decreasing inducible nitric oxide synthase (iNOS) and proinflammatory cytokines expression levels and conversely enhanced microglia M2 polarization by increasing Arginase-1 (Arg-1) and anti-inflammatory cytokine expression levels. These results strongly suggest that TREM2 provides a protective effect in VD via modulating the phenotype of activated microglia and may serve as a novel potential therapeutic target for VD.

## 1. Introduction

Vascular dementia (VD) describes a combination of the loss of cognitive functioning and memory associated with variable brain lesions of vascular origin [1]. As is well known, VD is widely considered as one of leading forms of dementia only after Alzheimer's disease (AD), accounting for 15-20% of all cases. With the advent of global aging, the incidence of VD is increasing steeply [2]. There are currently an estimated 50 million people living with dementia worldwide, and the number will rise to 82 million by 2030 and 150 million by 2050. VD poses a heavy financial burden on families and societies [3]. The global annual cost for dementia is expected to reach $2.54 trillion in 2030 and $9.12 trillion in 2050 [4]. Despite much progress on VD research over the past several decades, the exact mechanism still remains obscure. Thus, it is imperative to determine the etiology of VD and search for an effective treatment.

The triggering receptor expressed on myeloid cells 2 (TREM2) protein is a type I transmembrane innate immune receptor of the TREM family. TREM2 is expressed exclusively by myeloid cells, and in the brain, TREM2 is predominantly expressed in microglia. TREM2 has been implicated in a wide range of functions including cell proliferation, phagocytosis, maturation, and inflammatory response [5]. Recently, several studies have also shown that TREM2 plays an important role in microglia cell activation and survival [6]. Microglia are one of the main cell types which are involved in the inflammatory responses in the central nervous system [7]. However, microglia-induced inflammation is a double-edged sword, which has both beneficial and detrimental effects on neurons according to different diseases and status.

Neuroinflammation is defined as activation of the innate immune system in response to different brain injuries. Microglial activation in the brain parenchyma is the hallmark of neuroinflammation and is thought to be a critical determinant of neuronal fate [8]. Neuroinflammation is closely related to the pathogenesis of various cerebrovascular diseases including VD [9]. Though TREM2 has been widely reported to regulate neuroinflammation, its role in VD has rarely been reported. In our previous study, we found that serum levels of soluble TREM2 are lower in VD patients than in healthy controls and TREM2 may be a potential predictive biomarker of cognitive decline in VD [10]. The purpose of our present study was to determine whether TREM2 plays a neuroprotective role by regulating inflammation in a mouse model of VD. The neuroprotective role of TREM2 in VD, if confirmed, may represent a potential therapeutic target for VD.

## 2. Materials and Methods

### 2.1. Animals

Adult male C57BL/6 mice (8-10 weeks old, purchased from Shanghai SLAC Laboratory Animal Co., Shanghai, China) were used for the experiments. All mice were accommodated in a controlled environment and free access to water and food. Animals were accommodated in steel cages under standard housing conditions in a room kept at 22 ± 1°C with a 12 h light, 12 h dark cycle. All animal experimental procedures were performed in accordance with the approved animal protocols and guidelines established by Shandong First Medical University & Shandong Academy of Medical Sciences.

### 2.2. Mouse Model of VD Induction

The transient bilateral common carotid artery occlusion (BCCAO) surgery was performed as previously described with minor modifications [11]. Briefly, mice were anesthetized with 2% isoflurane in 30% oxygen, then both the right and left common carotid arteries were isolated from the adjacent vagus nerve and a silk was passed below each carotid artery for closure. The bilateral carotid arteries were locked by silk strings for 15 min and then released for 15 min, and this was repeated three times. The strings were then removed and the incision was sutured. Throughout the experiment, mice were placed in an automatic temperature-controlled chamber (World Precision Instruments, Sarasota, Florida, USA) to keep their body temperature at 37°C. After surgery, the mice were then moved to their original cages after 2 h and allowed to recover for 24 h before the start of subsequent operation.

### 2.3. Experimental Groups

Following 24 h of recovery from surgery, mice were randomly divided into the following three groups with 10 animals in each group. (1) Sham group: mice were given the same surgical procedure without carotid artery occlusion and the control lentivirus (LV-control) was injected into the right lateral ventricle; (2) VD+LV-control group: mice were subjected to the transient BCCAO modeling surgery and the control lentivirus (LV-control) was injected into the right lateral ventricle; (3) VD+LV-TREM2 group: mice were subjected to the transient BCCAO modeling surgery and the lentivirus overexpressing TREM2 (LV-TREM2) was injected into the right lateral ventricle.

### 2.4. Lentiviral Vector Preparation and Administration

The lentiviral vector that encoded TREM2 and control lentiviral vector were prepared by GENECHEM Biotech. Co. Ltd. (Shanghai, China). Stereotactic intracerebral injection of lentiviral vector was conducted by technicians who were blinded to the experimental groups as previously described [12, 13]. Briefly, mice were anesthetized and fixed on a stereotactic frame, then the lentiviral vector was injected into the right lateral ventricle (stereotaxic coordinates: 1.0 mm near the midline, 0.2 mm posterior to the bregma, and 3.0 mm below the skull). The injections were performed in a volume of 2 *μ*l for 5 min, and the infusion microsyringe (Hamilton, Reno, NV) was maintained for diffusion for an additional 5 min.

### 2.5. Morris Water Maze Test

Four weeks after the intracerebroventricular administration, cognitive deficits were assessed by the Morris water maze (MWM JK001 type, Beijing, China), which was performed as previously described [12, 14]. The MWM apparatus consists of a black cylindrical pool (diameter:150 cm; height:60 cm), a video camera, and a computerized system (EthoVision, Version 8.5, Noldus Information Technology, Wageningen, the Netherlands). The MWM was filled with water at 24 ± 1°C, which was divided into four quadrants and made opaque by the addition of skim milk powder. The MWM test included an acquisition training phase and a probe phase to assess memory. All the data was measured by an automated analysis system.

In the acquisition training phase, the escape platform with a diameter of 10 cm was fixed in the center of one quadrant and submerged 1 cm beneath the water surface. The acquisition training phase was repeated for four consecutive days, and a total of four trials were conducted per day. In each trial, the mice were released from the four quadrants, respectively, and given a 90 s (max) to find the submerged platform. If the mice could not find the platform in 90 s, the mice were guided onto the platform. The mice were allowed to remain on the platform for 30 s. The swim speed and the swimming time to the hidden platform (escape latency) were recorded.

In the probe phase, the platform was removed from the pool. The mice were released from the four quadrants, respectively, and allowed to swim freely for 90 s. The time spent in the target quadrant (the quadrant time) and the frequency of crossing the target quadrant (passing quadrant times) were recorded.

### 2.6. Real-Time PCR

Total mRNA was harvested from mouse brain tissues using TRIZOL reagent (Invitrogen, Carlsbad, CA, USA) according to the manufacturer's instructions [15]. Synthesis of cDNA was performed using a ReverTra Ace qPCR RT kit (Toyobo Co., Osaka, Japan). The sequences of the specific primers for target genes are listed in Table 1. For reverse transcriptase qPCR assays, the SYBR Green Real-Time PCR Master Mix kit was used. The real-time PCR was conducted by ABI StepOnePlus Systems (Applied Biosystems, Foster City, CA, USA). The data of real-time PCR were analyzed as 2^-*ΔΔ*Ct^; *β*-actin was used as the internal control.

### 2.7. Western Blotting

Western blots were performed to measure the protein expression levels of TREM2, nitric oxide synthase (iNOS), and Arginase-1 (Arg-1) as previously described [16]. Protein was collected from brain tissues using RIPA buffer (50 mM Tris (pH 7.4), 150 mM NaCl, 1% NP-40, 0.5% sodium deoxycholate, 0.1% SDS) supplemented with protease inhibitors and Halt Phosphatase Inhibitor Mixture (Beyotime Biotech, Jiangsu, China). Protein extracts were denatured and subjected to 10% sodium dodecyl sulfate-polyacrylamide electrophoresis (SDS-PAGE, Beyotime Biotech, Jiangsu, China). After electrophoresis, protein was transferred onto a polyvinylidene difluoride (PVDF) (Millipore, Billerica, MA, USA) membrane. 5% fat-free milk was used to block the membranes for 2 h at room temperature and then incubated with primary antibodies (TREM2, iNOS, and Arg-1; ABclonal Biotech, Hubei, China) overnight at 4°C. After washing with TBST, the membranes were incubated with secondary antibody (Beyotime Biotech, Jiangsu, China) for 1 h at room temperature. The images were captured using Odyssey infrared fluorescence imaging system (Odyssey, LI-COR Bioscience, Lincoln, NE, USA).

### 2.8. Immunohistochemistry Assays

Immunohistochemistry (IHC) staining was performed to examine iNOS and Arg-1 protein expression in the hippocampal CA1 subregion in mice as previously described [17]. The hippocampal CA1 coronal sections were incubated with primary antibodies against iNOS and Arg-1 (1 : 200, ABclonal Biotech, Hubei, China) at 4°C overnight. Then, the sections were incubated with a secondary antibody (Beyotime Biotech, Jiangsu, China), followed by nucleus counterstaining stained with DAPI (1 : 1000, Sigma, St Louis, MO, USA) for 10 min. Microscopy (Olympus, Tokyo, Japan) was performed, and images were obtained at 40x. For all IHC experiments, control sections without primary antibodies were routinely used.

### 2.9. Nissl Staining

Nissl staining was used to detect neuronal injury as reported previously [18]. Three hippocampal CA1 coronal sections at different depths were imaged for each mouse, and three fields of the hippocampus on each coronal section were then randomly selected for quantitative analysis. Neurons with intact morphology and dark violet nucleus were identified as Nissl staining-positive neurons, and the numbers of Nissl staining-positive neurons were counted under a microscope (Olympus, Tokyo, Japan) by observers who were blinded to the experimental groups. The data are presented as the number of Nissl staining-positive neurons in the hippocampus.

### 2.10. Statistical Analysis

All data are represented as mean ± SD. The MWM data were analyzed by two-way repeated-measures analysis of variance (ANOVA). And other data were analyzed by one-way ANOVA followed by Tukey post hoc test. Statistical tests were performed using statistical software package SPSS version 22.0 (SPSS Inc., Chicago, IL), and *p* < 0.05 was considered significant differences.

## 3. Results

### 3.1. TREM2 Is Upregulated in a Mouse Model of VD

**Figure 1:**
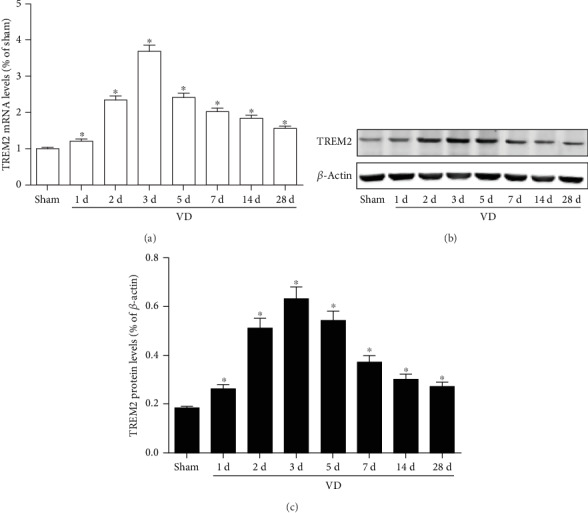
Expression of TREM2 in a mouse model of VD. (a) RT-PCR analysis of TREM2 mRNA level in the hippocampus at different time points of VD; (b, c) western blot analysis of TREM2 protein level in the hippocampus at different time points of VD. Compared to the sham group, ^∗^*p* < 0.05.

In order to study the role of TREM2 during VD pathogenesis, we first detected the time course of TREM2 expression in a mouse model of VD. We examined TREM2 gene and protein levels using RT-PCR and western blot, respectively. The mRNA level of TREM2 was upregulated in the hippocampus of VD mouse model, and the peak of TREM2 mRNA occurred three days after surgery (Figure 1(a)). TREM2 protein level, consistent with the mRNA level expression, showed the similar pattern (Figure 1(b)). Together, these results suggest that TREM2 is involved in the pathogenesis of VD.

### 3.2. TREM2 Overexpression Attenuates Cognitive Deficits in VD Mice

**Figure 2:**
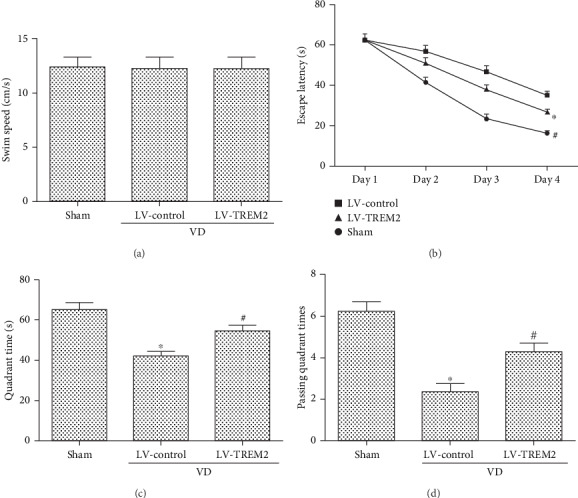
TREM2 overexpression attenuates cognitive deficits in VD mice: (a) swimming speed; (b) escape latency; (c) quadrant time; (d) passing quadrant times. Compared to the sham group, ^∗^*p* < 0.05; compared to the LV-control group, ^#^*p* < 0.05.

To determine the therapeutic potential of TREM2, the lentiviral vector that encoded TREM2 was used in the lateral ventricle of VD mice and the MWM test was performed 4 weeks after the lentivirus injection. There was no statistical difference in swimming speed among the three groups (*p* > 0.05, Figure 2(a)). Compared with LV-control VD mice, LV-TREM2 VD mice showed reduced escape latency during the acquisition training phase (*p* < 0.05, Figure 2(b)). During the probe trial phase, the quadrant time (*p* < 0.05, Figure 2(c)) and passing quadrant times (*p* < 0.05, Figure 2(d)) were increased.

### 3.3. TREM2 Overexpression Modifies Microglia Phenotype in VD Mice

**Figure 3:**
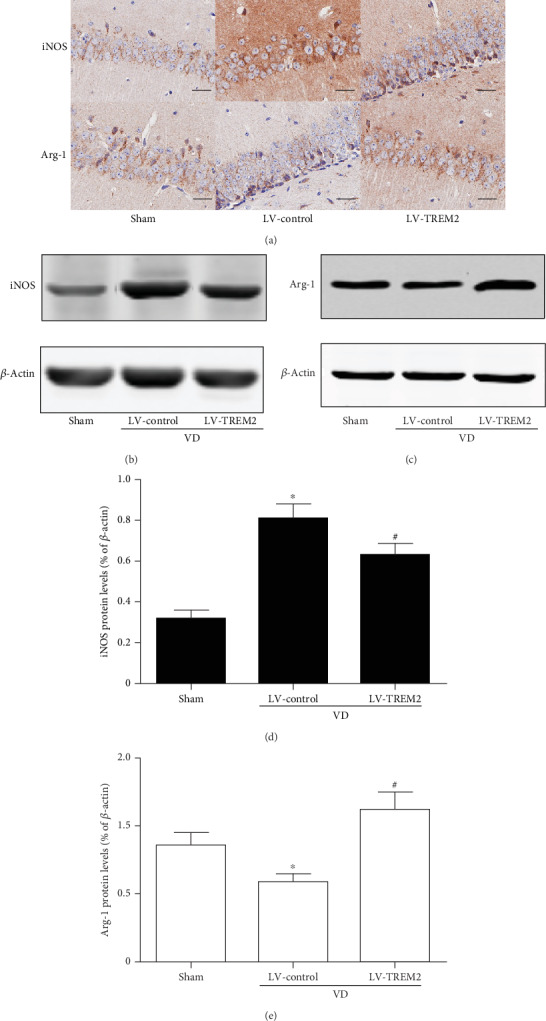
TREM2 overexpression modifies microglia phenotype in VD mice. (a) IHC staining of M1 microglia phenotype marker iNOS and M2 microglia phenotype marker Arg-1 in the hippocampus of VD mice. Scale bars = 50 *μ*m. (b–e) Western blot analysis of iNOS and Arg-1 protein level in the hippocampus of VD mice. Compared to the sham group, ^∗^*p* < 0.05; compared to the LV-control group, ^#^*p* < 0.05.

Microglia play different roles depending on its phenotype in the progression of many diseases including VD. It is generally believed that M1 phenotype microglia play a proinflammatory role, while M2 phenotype microglia play an anti-inflammatory role. In order to detect the effect of TREM2 on microglia phenotype, we used immunohistochemistry and western blotting to detect the expression of M1 microglia markers iNOS and M2microglia markers Arg-1 by TREM2 overexpression in VD mice. As indicated in Figure 3, TREM2 overexpression significantly reduced the protein levels of iNOS and increased the protein levels of Arg-1 (*p* < 0.05, Figures 3(a)–3(e)).

### 3.4. TREM2 Overexpression Attenuated Inflammatory Response in VD Mice

**Figure 4:**
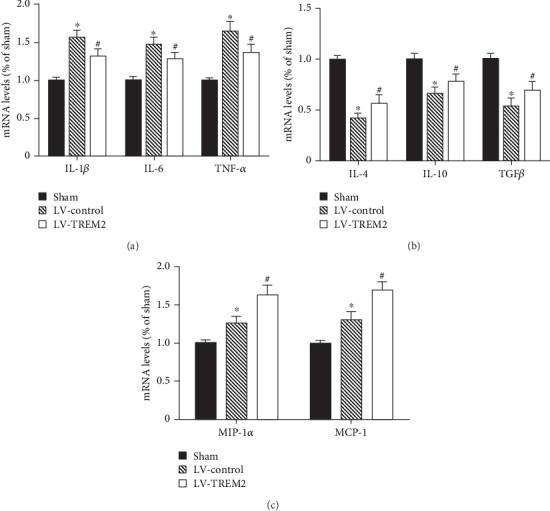
TREM2 overexpression attenuated inflammatory response in VD mice. (a) mRNA levels of proinflammatory mediators in the hippocampus of VD mice; (b) mRNA levels of anti-inflammatory mediators in the hippocampus of VD mice; (c) mRNA levels of chemokine cytokines in the hippocampus of VD mice. Compared to the sham group, ^∗^*p* < 0.05; compared to the LV-control group, ^#^*p* < 0.05.

To examine the effect of TREM2 overexpressed on inflammatory response, mRNA levels of proinflammatory mediators (IL-1*β*, IL-6, and TNF-*α*), anti-inflammatory mediators (IL-4, IL-10, and TGF*β*), and chemokine cytokines (MIP-1*α*, MCP-1) were measured by RT-PCR in VD mice. The proinflammatory mediators and anti-inflammatory mediators are considered secretions of the M1 and M2 phenotype microglia, respectively. We found that TREM2 overexpression significantly downregulated mRNA levels of proinflammatory mediators (*p* < 0.05, Figure 4(a)), while it significantly upregulated mRNA levels of anti-inflammatory mediators (*p* < 0.05, Figure 4(b)) and chemokine cytokines (*p* < 0.05, Figure 4(c)).

### 3.5. TREM2 Overexpression Prevents Neuronal Loss in VD Mice

**Figure 5:**
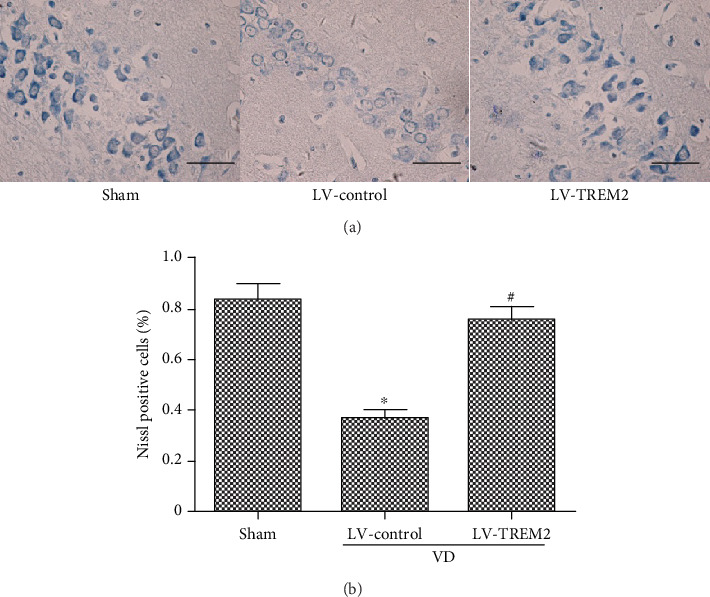
TREM2 overexpression prevents neuronal loss in VD mice. (a) Nissl staining was performed to detect neuronal loss in the hippocampus of VD mice. (b) The percentage of Nissl-positive neurons in the hippocampus of VD mice. Scale bars = 50 *μ*m. Compared to the sham group, ^∗^*p* < 0.05; compared to the LV-control group, ^#^*p* < 0.05.

Loss of neurons in the hippocampus is associated with cognitive deficits. In order to detect neuronal injury, we observed hippocampal neurons with Nissl staining. As indicated in Figure 5, TREM2 overexpression significantly attenuated neuronal loss in the hippocampus of VD mouse (*p* < 0.05, Figures 5(a) and 5(b)).

## 4. Discussion

In the present study, our results showed that TREM2 gene and protein levels were upregulated in the brains of VD mice compared with the sham mice, indicating that TREM2 may be involved in the pathogenesis of VD, which was consistent with our previous study [10]. Further investigation revealed that TREM2 overexpression attenuates cognitive deficits and neuronal loss. Moreover, TREM2 may regulate the release of inflammatory factors by modifying the microglia phenotype, which may partly explain the protective effects of TREM2 against cognitive deficits and neuronal loss in the mouse models of VD. To our knowledge, our present study is the first to elucidate the important role of TREM2-mediated microglial phenotypic polarization and inflammatory response in the pathogenesis of VD.

Accumulating evidence suggests that inflammation is a major contributor in the pathogenesis of many neurological diseases including VD. Neuroinflammation in VD is characterized by elevated levels of inflammatory mediators released from activated microglia [19]. In an animal study, Sun and his colleagues found that Rehmannioside A could attenuate cognitive deficits in rats with VD through reducing the release of proinflammatory cytokines, including TNF-*α*, IL-1*β*, and IL-6 [20]. Several other drugs, such as resveratrol, cannabinoid receptor agonist, and vanillic acid, have also been shown to play a neuroprotective role in different animal models of VD by reducing the inflammatory response [21–23]. Interestingly, a study suggests that acupuncture, a traditional Chinese treatment, may also improve cognitive function by reducing neuroinflammation [24]. The above animal studies suggest that inflammation is involved in the pathogenesis of vascular dementia. Apart from animal models, the important role of inflammation in the pathogenesis of VD has also been found in clinical studies. We previously found that *Helicobacter pylori* may aggravate atherosclerosis and cognitive impairment in VD patients by increasing the serum levels of inflammatory mediators [25, 26]. Although the role of inflammation in VD has been widely reported, its upstream and downstream mechanisms have not been fully elucidated.

Recently, several studies have revealed that TREM2 is involved in the inflammatory pathology of a variety of neurological disorders [27, 28]. One study showed that LPS-treated APP/PS1 transgenic mice had decreased TREM2 levels and increased TLR4 levels, indicating that TLR4/TREM2 may be a potential link between AD and systemic inflammation and TREM2 can serve as a potential therapeutic target for treating systemic inflammation in AD [29]. Another study revealed that TREM2 inhibited the activation of TNF-*α-*induced inflammation response in rheumatoid arthritis via the p38 pathway [30]. Moreover, the studies of Ren et al. suggested that TREM2 has a neuroprotective effect in Parkinson's disease by reducing neuroinflammation and the apoptosis of dopamine neurons [31]. Taken together, TREM2 may be an upstream regulator of inflammation in a variety of neurological disorders.

Neuroinflammation is primarily driven by microglia, which are the innate immune cells of the central nervous system and play a central role in many aspects of brain metabolism and physiology [32]. There are two main types of activated microglia: the proinflammatory M1 phenotype and the anti-inflammatory M2 phenotype. Different activated statuses of microglia secrete completely different arrays of cytokines. On the one hand, microglia cause neuronal injury via secreting proinflammatory cytokines, and on the other hand, they execute beneficial effects via releasing anti-inflammatory mediators. Thus, microglia are considered a double-edged sword. In recent years, the relationship between TREM2 and microglia has attracted much attention [33]. One study found that TREM2 modified microglial phenotype in P301S tau transgenic mice [34]. The other study demonstrated solution TREM2 against attenuated amyloid pathology and related toxicity, suggesting that TREM2 plays a neuroprotective role in the body and can be explored as a therapeutic target for AD [35]. A study found that TREM2 has the potential to maintain endothelial cell homeostasis as a microglial receptor and signaling hub, suggesting an underlying link between immune response and vascular disease [36]. However, the involvement of TREM2-mediated neuroinflammation in the pathogenesis of VD has not been previously reported.

In our previous study, we found that the expression of serum soluble TREM2 was decreased and serum soluble TREM2 levels were an independent risk factor for cognitive impairment in VD patients [10]. However, the results of our current study showed that the gene and protein levels of TREM2 were elevated in VD model mice. The two results seem conflicting. I think there are two reasons for this difference. The one is the different periods of inflammation. Acute inflammation is thought to have a protective effect on the body, while chronic inflammation is thought to be harmful [8]. The inflammation in VD patients is mostly in the chronic phase, whereas the inflammation in VD mice is in the acute phase. In the acute inflammatory phase of VD mice, higher TREM2 levels may be an organism's self-protection mechanism. The other is the different molecular structure and localization. Solution TREM2 (sTREM2) is a proteolytic product of TREM2 released to the extracellular space, which can be detected in the serum [37]. Assume that the total amount of sTREM2 and TREM2 is constant, there may exist a certain dynamic equilibrium between sTREM2 in the serum and TREM2 in the brain parenchyma. These two differences might partly explain the conflicting outcomes. In future studies, we will detect levels of sTREM2 in the peripheral blood of VD mice and investigate the different roles of sTREM2 and TREM2 in the pathogenesis of VD.

## 5. Conclusion

Taken together, in our current study, we provide novel evidence that TREM2 overexpression attenuates cognitive deficits and neural loss through modulating the phenotype of activated microglia and reducing inflammatory reaction. These findings shed new light on the role of TREM2 in the pathogenesis of VD and indicate that TREM2 may represent a promising novel target for VD. The relationship between TREM2 and VD, if confirmed by future large multicenter studies, may have crucial clinical and therapeutic implications.

## Figures and Tables

**Table 1:** Sequences of primers used in real-time PCR.

Gene name	Forward primer (5′-3′)	Reverse primer (5′-3′)	Accession number	Size (bp)
TREM2	ACAGCACCTCCAGGAATCAAG	AACTTGCTCAGGAGAACGCA	NM_031254.3	82
IL-1*β*	TGCCACCTTTTGACAGTGATG	TGATGTGCTGCTGCGAGATT	NM_008361.4	138
IL-6	GACAAAGCCAGAGTCCTTCAGA	TGTGACTCCAGCTTATCTCTTGG	NM_001314054.1	76
TNF-*α*	GATCGGTCCCCAAAGGGATG	CCACTTGGTGGTTTGTGAGTG	NM_001278601.1	92
IL-4	CCATATCCACGGATGCGACA	AAGCACCTTGGAAGCCCTAC	NM_021283.2	166
IL-10	GCTCTTGCACTACCAAAGCC	CTGCTGATCCTCATGCCAGT	NM_010548.2	112
TGF*β*	AGGGCTACCATGCCAACTTC	CCACGTAGTAGACGATGGGC	NM_011577.2	168
MIP-1*α*	TCTGCGCTGACTCCAAAGAG	CTCAAGCCCCTGCTCTACAC	NM_011337.2	130
MCP-1	TGCCCTAAGGTCTTCAGCAC	AAGGCATCACAGTCCGAGTC	NM_011333.3	150

## Data Availability

The data used to support the findings of this study are available from the corresponding author upon reasonable request.
